# Comparison of circulating tumor cells and AR-V7 as clinical biomarker in metastatic castration-resistant prostate cancer patients

**DOI:** 10.1038/s41598-022-16094-6

**Published:** 2022-07-13

**Authors:** Katrin Schlack, Konstantin Egon Seifert, Neele Wüstmann, Verena Humberg, Norbert Grundmann, Julie Steinestel, Dorothee Tiedje, Kambiz Rahbar, Laura-Maria Krabbe, Martin Bögemann, Andres J. Schrader, Christof Bernemann

**Affiliations:** 1 Research Lab, Department of Urology, University Hospital Muenster, Albert-Schweitzer Campus 1 A1, 48149 Muenster, Germany; 2 Institute for Bioinformatics, University Hospital Muenster, Muenster, Germany; 3 Department of Urology, University Hospital Augsburg, Augsburg, Germany; 4 Department of Nuclear Medicine, University Hospital Muenster, Muenster, Germany

**Keywords:** Predictive markers, Prognostic markers

## Abstract

Biomarker in metastatic castration resistant prostate cancer (mCRPC) treatment are rare. We aimed to compare the clinical value of circulating tumor cells (CTCs) and androgen receptor splice variant 7 (AR-V7) as biomarker in mCRPC patients undergoing androgen receptor-targeted agent (ARTA) treatment. Overall cohort (65 patients) was stratified regarding either CTC or AR-V7 status followed by further sub-stratification of the respective other marker. Subsequently, prostate specific antigen (PSA) response, progression free survival (PFS) and overall survival (OS)) of subgroups was compared. CTCs and AR-V7 were detected in 54 (83%) and 33 (61%) patients, respectively. All AR-V7 + were CTC +. We detected PSA response in all subgroups. For PFS and OS, biomarker stratification revealed differences between all subgroups. Interestingly, no significant differences of AR-V7 transcript copy numbers were detected between responding and non-responding patients. Additionally, multivariable analysis revealed no independent prognostic value of AR-V7 positivity. Both biomarkers show clinical value in prognosticating clinical outcome. Nonetheless, AR-V7 stratification underestimates the heterogenous subgroup of CTC − and CTC + patient, the latter requiring more intense clinical surveillance. Additionally, AR-V7 level does not correlate with clinical response. Thus, the value of AR-V7 as a clinical biomarker must be considered skeptically.

## Introduction

Studies on circulating tumor cells (CTCs) have significantly increased knowledge of dissemination of tumor cells from primary and metastatic tumor sites^1^. Large patient analyses of CTCs revealed a prognostic threshold of CTCs in prostate cancer^2,3^.

Besides analysis of CTCs, expression of androgen receptor splice variant 7 (AR-V7) in CTCs of prostate cancer patients has gained clinical interest for prediction of non-response to androgen receptor-targeted agents (ARTAs), i.e., abiraterone or enzalutamide^4–7^. However, there are inconsistencies among study designs analyzing the correlation between AR-V7 and treatment response. Initially, correlation analysis was performed solely in CTC + patients, further subdivided into AR-V7- and AR-V7 + subgroups^4^. A follow-up study then included CTC − patients, resulting in three groups (CTC −, CTC +/AR-V7- and CTC +/AR-V7 + patients)^7^. Further studies stratified AR-V7- and AR-V7 + only, without determination of the CTC status^6,8–12^. Of note, all studies showed a subset of AR-V7- patients not responding to ARTA treatment, demonstrating additional resistance mechanisms other than AR-V7.

Thus, both, CTCs as well as AR-V7, have been demonstrated to be potential clinical biomarkers. Nonetheless, both stratification approaches lack the integration of the other marker. Therefore, we aimed to comprehensively compare the risk assessment of CTC and AR-V7 stratification on clinical outcome in a defined cohort of mCRPC patients undergoing ARTA treatment.

## Material and methods

### CTC enrichment and determination

Blood sample preparation and CTC analysis have been described previously^13,14^. Briefly, blood samples of mCRPC patients (5 ml of each patient) were processed using the Dynabeads™ Epithelial Enrich Kit followed by mRNA isolation using the Dynabeads™ mRNA DIRECT™ Purification Kit (both ThermoFisher Scientific, Waltham, MA, USA). Complementary DNA (cDNA) synthesis was performed using the SuperScript™ IV VILO™ Master Mix (ThermoFisher Scientific, Waltham, MA, USA) according to manufacturer’s recommendation, except for using a temperature of 55 °C.

For determination of CTCs, patient samples were processed followed by qPCR using a KLK3-PSA TaqMan assay (Hs03063374_m1) (ThermoFisher Scientific, Waltham, MA, USA). A patient sample was determined CTC + when displaying a qPCR signal for KLK3-PSA. Given the nature of our CTC detection system, we are not able to determine the actual number of CTCs. However, patients display similar expression levels of KLK3-PSA in single CTCs^15^. Thus, determination of KLK3-PSA transcript copy numbers can be used as a semi-quantitative tool to dissect CTC distribution. AR-V7 status was analyzed using a previously described custom-made AR-V7 TaqMan PCR assay, demonstrating identical performance compared to the Johns Hopkins University (JHU) AR-V7 assay^14^. All qPCR runs were performed along with TaqMan qPCR assays for housekeeping genes RPL37A (Hs01102345_m1) and HPRT1 (Hs99999909_m1) (ThermoFisher Scientific, Waltham, MA, USA).

### Patient subgrouping

**Figure 1:**
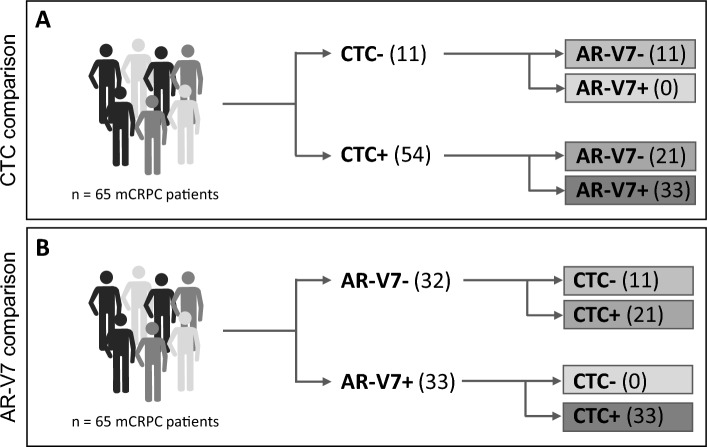
Patient subgrouping according to biomarker status. For comparative analysis of either CTC (**A**) or AR-V7 (**B**) as clinical biomarker, the overall cohort was stratified according to the respective biomarker status. Subsequently, subgroups were further subdivivded according to the respective other biomarker status. Colored boxes indicate identical subgroups.

For comparative biomarker stratification and clinical outcome analyses the overall cohort was sub-grouped based on either KLK3-PSA mRNA detection as a surrogate for CTCs or AR-V7 mRNA expression into CTC − and CTC + as well as AR-V7- and AR-V7 + groups, respectively. The CTC + subgroup was further subdivided into CTC +/AR-V7- and CTC +/AR-V7 + patients. Also, the AR-V7- subgroup was further subdivided into CTC −/AR-V7- and CTC +/AR-V7- patients (Fig. 1).

### Outcome measures

The primary endpoint was clinical or radiographic PFS. The assessment of response status took place at each routinely planned visit and was independently performed by experienced experts in the field of mCRPC. For the classification of response status, general condition (ECOG), presence of pain, laboratory constellations (mainly PSA) as well as imaging were considered. Clinical progression was defined as symptomatic progression (worsening or new prostate cancer-related symptoms). PSA progression was defined as confirmed increase of 25% or greater from baseline in case of no prior PSA decline and an absolute increase of 2 ng/ml or more from nadir (Prostate Cancer Working Group 3 criteria). During clinically and biochemically stable disease, imaging consisting of CT- or MRI-scans of thorax, abdomen, and pelvis as well as bone scans, was not performed routinely. When biochemical progression occurred or PD was otherwise presumed, soft tissue metastases were evaluated by CT- and MRI-scans and bone metastases were assessed by bone scans. For all patients, PD was defined according to RECIST 1.1 criteria for cross-sectional imaging and by PCWG3 criteria for bone scans.

Secondary endpoints included PSA response and OS. PSA response was defined as a PSA decline of 50% or more from baseline at any time under therapy; best PSA response (maximum percentage decrease from baseline) was also determined. OS was defined as the interval from start of ARTA to death from any cause.

### Statistical analyses

Statistical analyses were performed using SPSS-Statistics V28.0 (IBM Inc., Armonk, NY, USA) and Prism 8 V8.4.3 (GraphPad Software, LLC., San Diego, CA, USA). The descriptive statistics are reported as medians with interquartile ranges (IQR). Uni- and multivariate analyses were done by Cox-regression-models. Hazard ratios (HR) are given with 95% confidence intervals (CI). All reported *p* values are two-sided and statistical relevance was assumed with a *p* value > 0.05.

Further detailed information can be found in the Supplementary Material.

### Ethics approval and consent to participate

The study was approved by the local Ethics committee (Ethik-Kommission der Ärztekammer Westfalen-Lippe und der Westfälischen Wilhelms-Universität; 2007-467-f-S and 2016–585-f-S) and all patients provided written informed consent. The study was conducted with provisions of the Declaration of Helsinki.

### Consent for publication

The authors agree the terms of conditions for publication.

## Results

### Patients

Between June 2016 and December 2020, we prospectively enrolled 65 patients who started on either abiraterone (n = 46) or enzalutamide (n = 19). At the time of study closure in March 2021, the median follow-up was 14.0 (IQR 8–31) months. 33 (50.8%) patients started as first-line treatment, 15 (23.1%) patients as a second-line treatment and 17 (26.2%) patients were treated in a third- or higher line of treatment (Table 1). All baseline characteristics are displayed in Table 1.

**Table 1:** Baseline characteristics of patients with mCRPC starting treatment with abiraterone or enzalutamide.

	Total
Patients [n]	65
Age, median [years] (IQR)	66 (62–76)
**Therapy [n] (%)**	
Abiraterone	46 (70.8)
Enzalutamide	19 (29.2)
Prior abiraterone or enzalutamide [n] (%)	22 (33.9)
Prior use of docetaxel [n] (%)	25 (38.5)
**Line of therapy [n] (%)**	
1st	33 (50.8)
2nd	15 (23.1)
3rd or higher	17 (26.2)
PSA doubling time < 3 months [n] (%)	28 (43.1)
**ECOG performance status (all) [n] (%)**	
0	53 (94.6)
≥ 1	3 (5.4)
Gleason-Score ≥ 8 [n] (%)	47 (77.1)
Median PSA at baseline [ng/ml] (IQR)	24.3 (9.3–114.0)
Median LDH at baseline [U/l] (IQR)	223 (197–275)
Median ALP at baseline [U/l] (IQR)	106 (76–169)
Hb < 12 g/dl [n] (%)	21 (32.3)
Elevated LDH at baseline [n] (%)	27 (41.5)
Elevated ALP at baseline [n] (%)	21 (32.3)
Bone protection [n] (%)	14 (21.5)
Presence of lymph node metastases [n] (%)	37 (56.9)
Presence of bone metastases [n] (%)	51 (78.5)
Presence of visceral metastases [n] (%)	6 (9.2)

*ALP* alkaline phosphatase, *ECOG* eastern co-operative oncology group, *Hb* hemoglobin, *IQR* interquartile range, *LDH* lactate dehydrogenase, *PSA* prostate specific antigen.

Eleven (17.0%) patients of the overall cohort were found to be negative for KLK3-PSA expression, i.e., absence of CTCs, whereas 54 (83.0%) patients were found to be positive for CTCs. We did not notice AR-V7 expression in any CTC − patient. Within the subgroup of CTC + patients, we detected AR-V7 in 33 (61.1%) patients (CTC +/AR-V7 +) whereas 21 (38.9%) patients did not display AR-V7 expression (CTC +/AR-V7-). In the AR-V7- subgroup, 11 patients (33.3%) displayed absence of CTCs, 21 patients were found to be positive for CTCs (66.7%).

### PSA response

**Figure 2:**
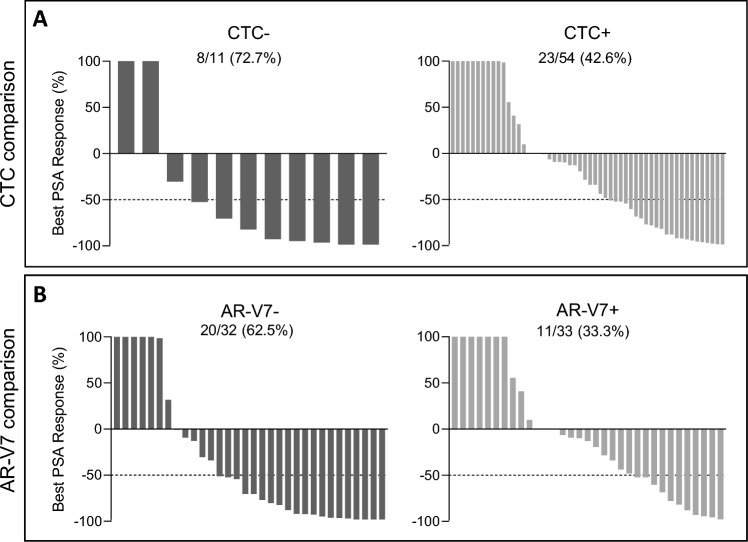
PSA response. Waterfall plots depicting best PSA responses in patients starting ARTA treatment according to CTC status, (**A**, left panel) CTC − patients, (**A**, right panel) CTC + patients, and AR-V7 status, (**B**, left panel) AR-V7- patients, (**B**, right panel) AR-V7 + patients. The dotted line illustrates the threshold of PSA 50% decline defining a PSA response. When comparing patients concerning CTC status, PSA response rates were 8/11 (72.7%) and 23/54 (42.6%) in CTC − and CTC + patients, respectively, without statistical significance (*p* = 0.10; Fisher’s exact test). PSA response rates were 20/32 (62.5%) and 11/33 (33.3%) in AR-V7- and AR-V7 + patients, respectively (*p* = 0.03; Fisher’s exact test).

We observed a PSA decline ≥ 50% (PSA response) in 31 (47.7%) patients of the overall cohort. Compared to the CTC + group (23/54 (42.6%)), more CTC − patients showed a PSA response (8/11 (72.7%; *p* = 0.10) (Fig. 2A). When comparing the AR-V7 + versus AR-V7- group, we detected a PSA response in 11 (33.3%) of the AR-V7 + patients and 20 (62.5%) of the AR-V7- patients (*p* = 0.03) (Fig. 2B).

### Progression free survival (PFS)

**Figure 3:**
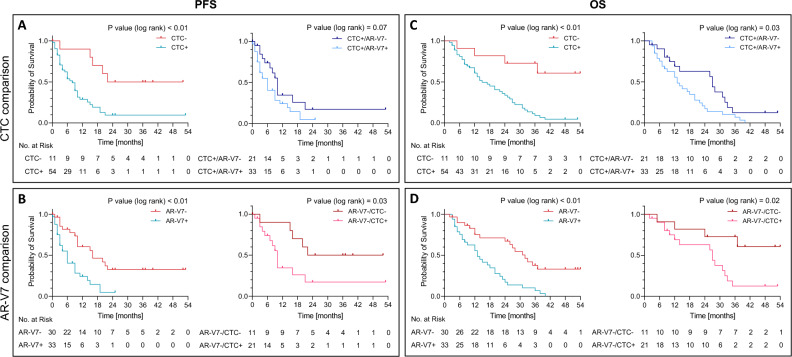
Progression free survival and overall survival. Kaplan–Meier curves including patient numbers at risk indicating PFS according to CTC status (**A**) and AR-V7 status (**B**) of patients undergoing ARTA treatment. (**A**, left panel) For the overall cohort, median PFS was 22 months (CI not estimable) and 8 months (CI 5.5–10.5) for CTC − patients and CTC + patients, respectively (*p* < 0.01). (**A**, right panel) Within the CTC + cohort, median PFS was 10 months (CI 8.9–11.1) and 6 months (CI 3.6–8.4) for AR-V7- patients and AR-V7 + patients, respectively, without statistical significance (*p* = 0.07). (**B**, left panel) For the overall cohort, median PFS was 16 months (CI 7.0–25.0) and 6 months (CI 3.6–8.4) for AR-V7- patients and AR-V7 + patients, respectively (*p* < 0.01). (B, right panel) Within the AR-V7- subgroup, median PFS was 22 months (CI not estimable) and 10 months (CI 8.9–11.1) for CTC − patients and CTC + patients, respectively (*p* = 0.03). Kaplan–Meier curves including patient numbers at risk indicating OS according to CTC status (**C**) and AR-V7 status (**D**) of patients undergoing ARTA treatment. (**C**, left panel) For the overall cohort, median OS was not reached for CTC − patients and 15 months (CI 8.7–21.3) for CTC + patients (*p* < 0.01). (**C**, right panel) Within the CTC + cohort, median OS was 27 months (CI 25.1–28.9) and 13 months (CI 9.2–16.8) for AR-V7- patients and AR-V7 + patients, respectively (*p* > 0.01), without statistical significance (*p* = 0.07). (**D**, left panel) For the overall cohort, median OS was 31 months (CI 25.2–36.8) and 13 months (CI 9.2–16.8) for AR-V7- patients and AR-V7 + patients, respectively (*p* < 0.01). (**D**, right panel) Within the AR-V7- subgroup, median OS was not reached and 27 months (CI 25.1–28.9) for CTC − patients and CTC + patients, respectively, (*p* = 0.02).

For the overall cohort, median PFS was 9 months (CI 7.1–10.9). By comparing CTC + versus CTC − patients, PFS was significantly longer for CTC − patients with 22 months (CI not estimable) versus 8 months (CI 5.5–10.5) for CTC + patients (*p* < 0.01) (Fig. 3A, left panel). Within the CTC + subgroup, AR-V7- patients showed prolonged PFS (10 months (CI 8.9–11.1)) compared to AR-V7 + patients (6 months (CI 3.6–8.4)), however, without statistical significance (*p* = 0.07) (Fig. 3A, right panel).

By comparing AR-V7- and AR-V7 + patients, PFS was significantly longer for AR-V7- (16 months (CI 7.0–25.0) versus AR-V + patients (6 months (CI 3.6–8.4)) (*p* < 0.01) (Fig. 3B, left panel). Within the AR-V7- subgroup, CTC − patients displayed improved PFS (22 months (CI not estimable)) versus (10 months (CI 8.9–11.1)) in CTC + patients (*p* = 0.03) (Fig. 3B, right panel).

Performing univariate analysis for the overall cohort, prior abiraterone or enzalutamide (*p* < 0.01), Hb ≤ 12 g/dl at baseline (*p* = 0.01), absence of PSA decline ≥ 50% (*p* < 0.01), CTC positivity (*p* < 0.01) and AR-V7 positivity (*p* < 0.01) were statistically prognostic for PFS (Table S1). Within the CTC + subgroup, prior abiraterone or enzalutamide (*p* < 0.01), PSA doubling time < 3 months (*p* = 0.04) and absence of PSA decline ≥ 50% (*p* < 0.01) were significantly associated with worse PFS, whereas AR-V7 positivity did not show association with an inferior PFS (*p* = 0.09) (Table S2). Within the AR-V7- subgroup, prior abiraterone or enzalutamide (*p* < 0.01), absence of PSA decline ≥ 50% (*p* < 0.01) and CTC positivity (*p* < 0.05) were significantly associated with worse PFS (Table S3).

In multivariate cox regression analysis for the overall cohort, only prior abiraterone or enzalutamide (*p* = 0.01) was independently prognostic (Table S4). In CTC + patients, none of the parameters was independently prognostic (Table S5). Within the AR-V7- subgroup, prior abiraterone or enzalutamide (*p* = 0.02) was independently prognostic (Table S6).

### Overall survival (OS)

For the overall cohort, median OS was 20 months (CI 10.5–29.5). By comparing CTC + versus CTC − patients, median OS was not reached for CTC − patients compared to 15 months (CI 8.7–21.3) for CTC + patients (*p* < 0.01) (Fig. 3C, left panel). By comparing AR-V7- and AR-V7 + patients within the subgroup of CTC + patients, median OS was 27 months (CI 25.1–28.9) for AR-V7- patients and 13 months (CI 9.2–16.8) for AR-V + patients, displaying statistical significance (*p* = 0.03) (Fig. 3C, right panel).

By comparing AR-V7- and AR-V7 + patients, OS was significantly longer for AR-V7- patients (31 months (CI 25.2–36.8)) compared to AR-V7 + patients (13 months (CI 9.2–16.8)) (*p* < 0.01) (Fig. 3D, left panel). Within the AR-V7- subgroup, CTC − patients displayed improved OS (not reached) versus 27 months (CI 25.1–28.9) in CTC + patients (*p* = 0.02) (Fig. 3D, right panel).

Univariate analysis for the whole cohort showed that prior abiraterone or enzalutamide (*p* < 0.01), bone metastases (*p* = 0.03), ALP elevated at baseline (*p* < 0.01), Hb ≤ 12 g/dl at baseline (*p* < 0.01), absence of PSA decline ≥ 50% (*p* < 0.01), CTC positivity (*p* < 0.01), AR-V7 positivity (*p* < 0.01) were prognosticators of worse OS (Table S1).

Within the CTC + group of patients, prior docetaxel (*p* < 0.01), prior abiraterone or enzalutamide (*p* < 0.01), Hb ≤ 12 g/dl at baseline (*p* < 0.01), PSA doubling time < 3 months (*p* = 0.03) and AR-V7 positivity (*p* = 0.04) were prognosticators of inferior OS (Table S2). Within the AR-V7- subgroup, Hb ≤ 12 g/dl at baseline (*p* < 0.01) and CTC positivity (*p* = 0.03) were significantly associated with worse OS (Table S3).

In multivariable cox-regression-analysis for the overall cohort, CTC positivity (*p* = 0.04), prior abiraterone or enzalutamide (*p* = 0.02) and Hb ≤ 12 g/dl at baseline (*p* < 0.01) were independent prognosticators for worse OS (Table S4). For CTC + patients, in multivariate analysis, only Hb ≤ 12 g/dl at baseline (*p* < 0.01) was independently prognostic for worse OS (Table S5). Within the AR-V7- subgroup, CTC positivity (*p* = 0.04) and Hb ≤ 12 g/dl at baseline (*p* < 0.01) were independent prognosticators for worse OS (Table S6).

### Quantification of biomarker transcripts and correlation to clinical outcome

For correlation analyses we determined KLK3-PSA and AR-V7 transcript copy numbers in CTC + and AR-V7 + patients, respectively, and stratified patients into responding (R) and non-responding (N) subgroups.

**Figure 4:**
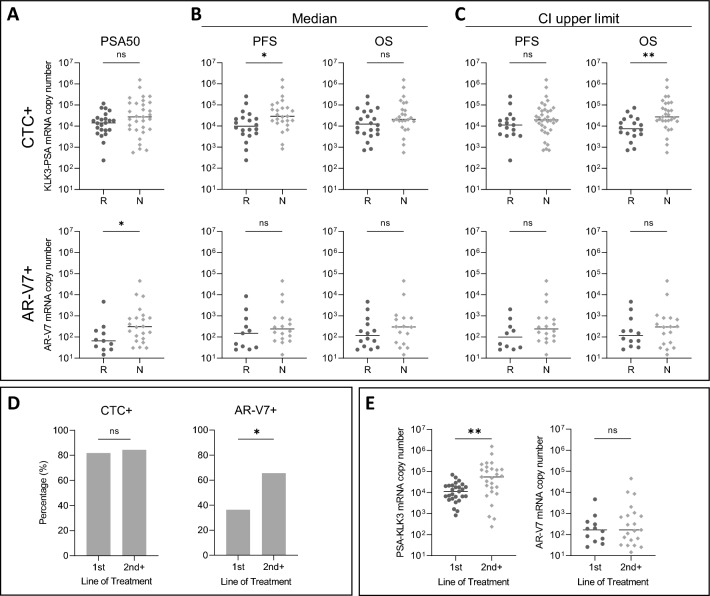
Correlation analysis of responding (R) and non-responding (N) patients and copy number distribution/biomarker appearance and levels. Comparison of mRNA copy numbers KLK3-PSA (upper panel) and AR-V7 (lower panel) per blood sample (5 ml) in biomarker positive patients (upper panel: CTC +; lower panel: AR-V7 +) regarding (**A**) PSA response, (**B**) PFS and OS categorized into responders ((R); PFS and OS duration > median PFS and OS) and non-responders ((N); PFS and OS duration < median PFS and OS) and (**C**) PFS and OS categorized into responders ((R); PFS and OS duration > CI upper limit for PFS and OS) and non-responders ((N); PFS and OS duration < CI upper limit for PFS and OS). *P* values (Mann–Whitney test) are as follows. CTC +: PSA50: *p* = 0.11; Median, PFS: *p* < 0.02, OS: *p* = 0.11; upper limit: PFS: *p* = 16, OS: *p* < 0.01; AR-V7 +: PSA50: *p* = 0.03; Median, PFS: *p* = 0.30, OS: *p* = 0.42; upper limit: PFS: *p* = 0.15, OS: *p* = 0.63. (**D**) Biomarker appearance among different lines of treatment. *P* values (Fisher’s exact test) are as follows. CTC +: *p* > 0.99; AR-V7 +: *p* = 0.03. (**E**) Biomarker levels among different lines of treatment. mRNA copy numbers of KLK3-PSA (as a surrogate for actual number of CTCs) and AR-V7 in different lines of treatment. *P* values (Mann–Whitney test) are as follows. KLK3-PSA: *p* > 0.01, AR-V7: *p* = 0.73.

For PSA response, no significant difference of KLK3-PSA transcript copy number was detected between responders and non-responders in the CTC + subgroup, whereas AR-V7 transcript levels were significantly lower in responding patients compared to non-responding patients (Fig. 4A). By applying the median PFS to differentiate between responders and non-responders, we detected a significantly higher number of KLK3-PSA transcript copy numbers within the subgroup of non-responders compared to responders, suggesting a worse clinical outcome in patients displaying a higher number of CTCs. However, no difference of KLK3-PSA transcript copy numbers between responders and non-responders was observed with respect to OS. No significant differences in PFS and OS were detected within the AR-V7 + subgroup (Fig. 4B).

We next aimed to perform a more conservative estimation by using the upper confidence interval as threshold to stratify patients into responders (above upper limit) and non-responders (below upper limit). By this, significant differences in KLK3-PSA transcript copy numbers were observed for both PFS and OS. However, for AR-V7, we did not detect any significant differences (Fig. 4C). This validates a correlation between a high number of CTCs and worse clinical outcome. Contrary, the expression level of AR-V7 does not influence clinical outcome.

### Association of CTC and AR-V7 status and advanced stage of disease

By dissecting distribution of biomarker positive patients (CTC + and AR-V7 +) among distinct lines of treatment, we detected a significant increase of AR-V7 + patients during disease progression, while the number of CTC + patients tend to stay similar (Fig. 4D). Contrary, the actual numbers of KLK3-PSA mRNA transcripts increase over different lines of treatment, whereas the number of AR-V7 mRNA transcripts did not show significant differences (Fig. 4E). This validates an increase in CTC numbers and AR-V7 appearance, yet no increase in transcriptional levels, over time.

We next intended to correlate biomarker subgroups and criteria of an advanced stage of disease (i.e., Hb, AP, LDH, presence of metastases, PSA doubling time). We detected a significantly higher number of criteria representing a more advanced stage of disease within both, CTC + compared to CTC − and AR-V7 + compared to AR-V7- subgroups (Fig. S1A). Whereas the count of criteria was similar in both, CTC + and AR-V7 + subgroups, a lower number of criteria was detected in the CTC − compared to the AR-V7- subgroup, yet without statistical significance (Fig. S1B).

## Discussion

In this study, we comprehensively compare the correlation of both, CTC and AR-V7 status, with clinical parameters in a cohort of mCRPC patients undergoing ARTA treatment. The results of our study indicate that both, CTC + versus CTC − and AR-V7 + versus AR-V7- stratification, can effectively prognosticate clinical outcome. Nonetheless, we demonstrate potential flaws by focusing on AR-V7 as a sole biomarker.

The presence of CTCs vividly holds the potential of a prognostic biomarker in mCRPC^16–20^. The number of more than 5 CTCs per 7.5 ml blood sample highly correlates with a worse prognosis^21^. Furthermore, CTC analyses not only determine CTC counts at baseline but might also be applicable during treatment. A decrease of CTC count of more than 30% during the first 12 weeks of treatment is associated with a good prognosis^19^. Also, PCWG3 guidelines recommend CTC counts as an endpoint in patients with unfavorable counts at baseline in clinical trials^22^. Our results validate the prognostic power of CTC determination by demonstrating a worse clinical outcome in CTC + versus CTC − patients with respect to PSA response, PFS and OS. When semi-quantifying the number of KLK3-PSA transcripts as a potential surrogate for actual number of CTCs, we detected a significant increase in transcript copy numbers in non-responding patients. This demonstrates the value of CTC number determination to prognosticate clinical outcome. Additionally, CTC positivity is associated with a more advanced stage of disease. Our results therefore underscore the validity of CTC detection approaches for clinical applications.

Besides, AR-V7 has been discussed as both, a prognostic and predictive biomarker in mCRPC. Initially, expression of AR-V7 has been correlated to non-response to ARTA in CTC + patients^4^. Although clinical outcome was worse in AR-V7 + patients, results might have been overestimated as AR-V7 + patients tend to suffer from an advanced stage of disease, e.g., higher number of previous treatments, higher serum PSA levels, and alkaline phosphatase levels. An extended follow-up study thus included CTC − patients and stratified patients into three categories: CTC −, CTC +/AR-V7- and CTC +/AR-V7 + ^7^. Within this study, significant differences were observed among the three subgroups, demonstrating the worst clinical outcome in CTC +/AR-V7 + patients. Consequently, the authors concluded, that AR-V7 analysis might be applied for prediction of ARTA treatment response. A biomarker-based application would imply clinical testing of AR-V7 status of all patients. Most recent studies then integrated dichotomous stratification of AR-V7 + or AR-V7- patients, without previous determination of CTC status^6,8–12^. In case of a strong predictive biomarker this stratification might be adequate. However, sole AR-V7 discrimination might be at risk of mis-consideration of severe clinical factors.

First, although PSA response is lower in AR-V7 + compared to CTC + patients, we show a substantial PSA response even in AR-V7 + patients. This is in line with previous reports demonstrating PSA response to ARTA even in AR-V7 + patients^11,23^. When focusing on the CTC + cohort, no significant differences in PFS were observed between CTC +/AR-V7- and CTC +/AR-V7 + patients. Additionally, we did not notice differences in AR-V7 transcript levels between patients responding and non-responding to ARTA treatment. Thus, the actual levels of AR-V7 seem not to correlate with either response or non-response. Hence, our results challenge the clinical value of AR-V7 as a predictive biomarker for ARTA treatment.

Second, in case of OS, we detected a significant difference between CTC +/AR-V7- and CTC +/AR-V7 + patients, demonstrating a prognostic value of AR-V7. However, for neither PFS nor OS, AR-V7 positivity was found to be an independent prognostic factor for both the overall cohort as well as the CTC + subgroup. Appearance of AR-V7 increases over different lines of treatment. Furthermore, AR-V7 positivity is associated with presence of criteria related to a later stage of disease as well as a more aggressive subtype of prostate cancer. These results are in line with a recent study by Sharp et al., demonstrating a correlation of AR-V7 and a more advanced stage of disease^24^. Given the lack of independent prognostic validity along with the fact, that a set of distinct criteria to stage patients into a high-risk subgroup, are already used in clinical routine, it remains unclear whether AR-V7 detection can add further information to improve patient treatment.

Third—and presumably of highest clinical impact, focusing on AR-V7 neglects the heterogeneity of the AR-V7- cohort consisting of both CTC − and CTC + patients. A sole focus on AR-V7 would thus overlook the heterogeneity of this subgroup of CTC + and CTC − patients. As discussed earlier, CTC + patients display a higher probability of worse prognosis compared to CTC − patients and thus, need accurate and frequent clinical surveillance. Indeed, we detected significant differences between AR-V7-/CTC − and AR-V7-/CTC + patients with respect to both PFS and OS. Additionally, in terms of OS, presence of CTCs is an independent prognostic factor for both the overall cohort and the AR-V7 + subgroup. Therefore, sole AR-V7 stratification is of risk of underestimating the risk of rapid disease progression in a subset of AR-V7- yet CTC + patients.

A limitation of this study is the relatively small number of included patients. Nonetheless, differences within the two biomarkers are obvious and significant.

Conclusively, our results demonstrate a prognostic value of mCRPC patient stratification regarding both, CTC and AR-V7 status. However, CTC status is clinically more meaningful in prognostication, whereas AR-V7 status is of risk of underestimating the clinical differences in AR-V7- patients by not discriminating between AR-V7-/CTC − and AR-V7-/CTC + patients. Ultimately, similar PFS outcomes of AR-V7 + and AR-V7- patients within the subgroup of CTC + patients strongly question the power of AR-V7 as a predictive biomarker for non-response to ARTAs. Thereby, our study underscores recent expert opinion neglecting the usage of AR-V7 for clinical decision making^25^.

## Supplementary Information

Supplementary Figure S1.

Supplementary Figure S2.

Supplementary Legends.

Supplementary Information.

Supplementary Tables.

## Data Availability

The datasets generated during and/or analysed during the current study are available from the corresponding author on reasonable request.

## Abbreviations

AR: Androgen receptor
AR-V7: Androgen receptor splice variant 7
ARTA: Androgen receptor-targeted agent
CTC: Circulating tumor cells
cDNA: Complementary DNA
CI: Confidence interval
HR: Hazard ratio
IQR: Interquartile range
JHU: Johns Hopkins University
mCRPC: Metastatic castration resistant prostate cancer
OS: Overall survival
PFS: Progression free survival
PSA: Prostate specific antigen
