# Supplementary figures and images for: Comparison of circulating tumor cells and AR-V7 as clinical biomarker in metastatic castration-resistant prostate cancer patients
Source: Sci Rep. 2022 Jul 13;12:11846. doi: 10.1038/s41598-022-16094-6 (PMC9279395; doi:10.1038/s41598-022-16094-6)

## Slide 1
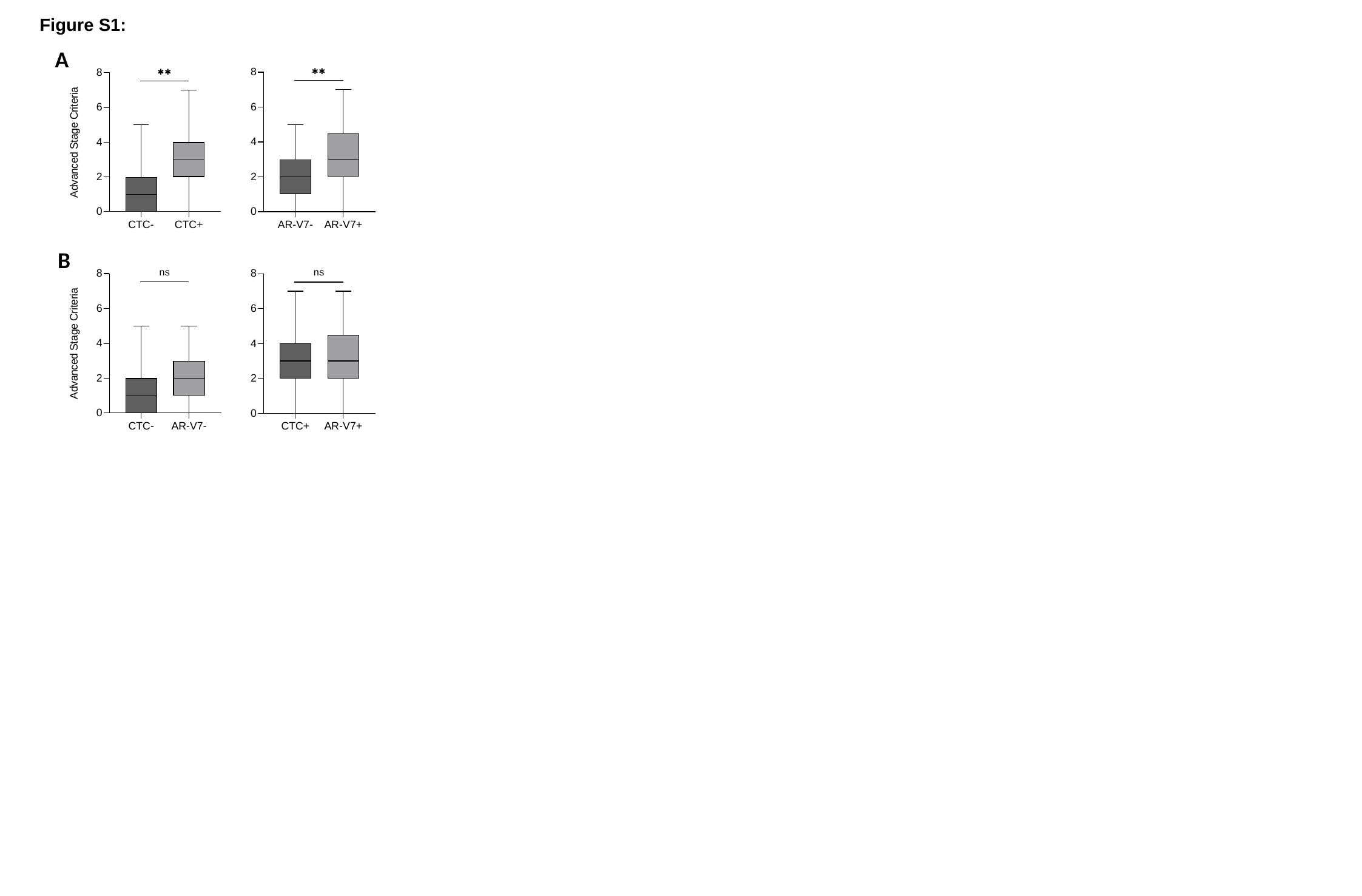

Figure S1:
A
B

Supplement: Supplementary file 1 — Supplementary Figure S1. [file 41598_2022_16094_MOESM1_ESM.pptx]
