# Supplementary material for: Comparison of circulating tumor cells and AR-V7 as clinical biomarker in metastatic castration-resistant prostate cancer patients
Source: Sci Rep. 2022 Jul 13;12:11846. doi: 10.1038/s41598-022-16094-6 (PMC9279395; doi:10.1038/s41598-022-16094-6)
Supplement: Supplementary file 2 — Supplementary Figure S2. [file 41598_2022_16094_MOESM2_ESM.pptx]

## Slide 1
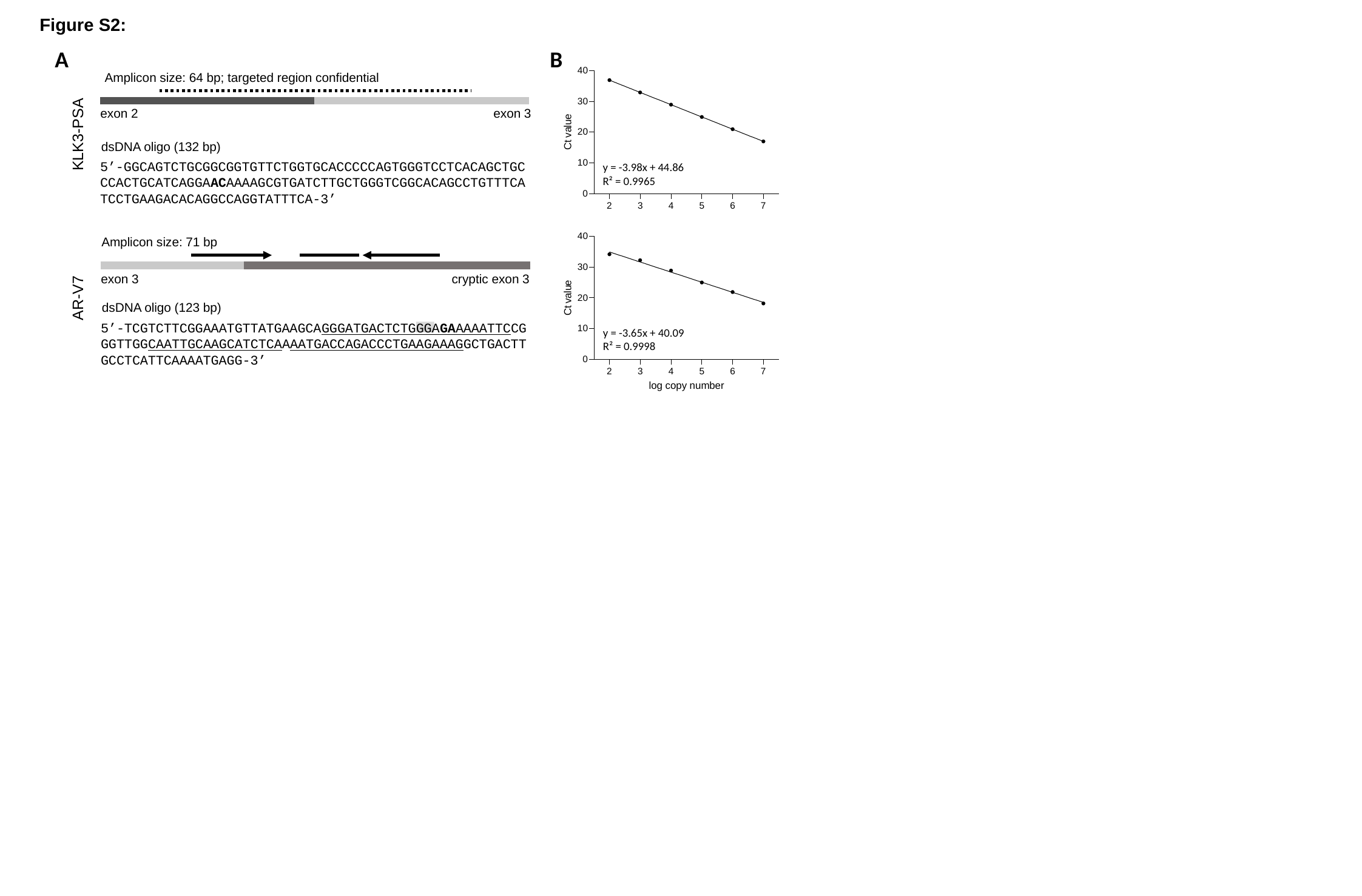

Figure S2:
A
B
Amplicon size: 64 bp; targeted region confidential
exon 3
exon 2
dsDNA oligo (132 bp)
5’‑GGCAGTCTGCGGCGGTGTTCTGGTGCACCCCCAGTGGGTCCTCACAGCTGCCCACTGCATCAGGAACAAAAGCGTGATCTTGCTGGGTCGGCACAGCCTGTTTCATCCTGAAGACACAGGCCAGGTATTTCA‑3’
KLK3-PSA
y = -3.98x + 44.86
R² = 0.9965
Amplicon size: 71 bp
cryptic exon 3
exon 3
dsDNA oligo (123 bp)
5’‑TCGTCTTCGGAAATGTTATGAAGCAGGGATGACTCTGGGAGAAAAATTCCGGGTTGGCAATTGCAAGCATCTCAAAATGACCAGACCCTGAAGAAAGGCTGACTTGCCTCATTCAAAATGAGG‑3’
AR-V7
y = -3.65x + 40.09
R² = 0.9998
