# Supplementary material for: Comparison of circulating tumor cells and AR-V7 as clinical biomarker in metastatic castration-resistant prostate cancer patients
Source: Sci Rep. 2022 Jul 13;12:11846. doi: 10.1038/s41598-022-16094-6 (PMC9279395; doi:10.1038/s41598-022-16094-6)
Supplement: Supplementary file 3 — Supplementary Legends. [file 41598_2022_16094_MOESM3_ESM.docx]

Supplementary figure legends and tables

**Fig. S1:** Association of advanced stage criteria and lines of treatment with biomarker appearance

(A) Box plots depicting comparison of advanced stage criteria in CTC- vs CTC+ and AR-V7- vs AR-V7+ patients. ** represent p-values <0.01 (B) Box plots depicting comparison of advanced stage criteria in biomarker negative (left panel) and biomarker positive (right panel) patients. No significant differences were observed (CTC- vs AR-V7- (p=0.24); CTC+ vs AR-V7+ (p=0.43); t test).

**Fig. S2:** mRNA copy number determination

(A) Overview of dsDNA oligonucleotides covering the spanning regions of KLK3-PSA and AR-V7 TaqMan qPCR assays. Dotted line displays region of KLK3-PSA assay (targeted sequence confidential); Arrows and line displays forward and reverse primers as well as hydrolysis probe. (B) Standard curves for KLK3-PSA and AR-V7 mRNA copy number quantification. Equations of curves were used to determine copy numbers per 5ml blood sample.

**Table S1**: Univariate analyses of biomarkers for PFS and OS in 65 mCRPC-patients on abiraterone or enzalutamide therapy (overall cohort)

**Table S2**: Univariate analyses of biomarkers for PFS and OS in 54 mCRPC-patients on abiraterone or enzalutamide therapy (CTC+ cohort)

**Table S3**: Univariate analyses of biomarkers for PFS and OS in 32 mCRPC-patients on abiraterone or enzalutamide therapy (AR-V7- cohort)

**Table S4**: Multivariate analyses of biomarkers for PFS and OS in 65 mCRPC-patients on abiraterone or enzalutamide therapy (overall cohort)

**Table S5**: Multivariate analyses of biomarkers for PFS and OS in 54 mCRPC-patients on abiraterone or enzalutamide therapy (CTC+ cohort)

**Table S6**: Multivariate analyses of biomarkers for PFS and OS in 32 mCRPC-patients on abiraterone or enzalutamide therapy (AR-V7- cohort)
