# Supplementary material for: Comparison of circulating tumor cells and AR-V7 as clinical biomarker in metastatic castration-resistant prostate cancer patients
Source: Sci Rep. 2022 Jul 13;12:11846. doi: 10.1038/s41598-022-16094-6 (PMC9279395; doi:10.1038/s41598-022-16094-6)
Supplement: Supplementary file 4 — Supplementary Information. [file 41598_2022_16094_MOESM4_ESM.docx]

**Supplementary Material**

Patients

For this study, we prospectively enrolled 65 men with mCRPC who presented at the Department of Urology at the University Hospital in Muenster, Germany between June 2016 and December 2020 starting treatment with either enzalutamide or abiraterone. All patients had histologically confirmed adenocarcinoma of the prostate and progressive disease (PD). PD was defined as either biochemical or radiographic progression according to Prostate Cancer Working Group 3 guidelines. Prior taxane chemotherapy was allowed, as well as previous treatment with the alternative antihormonal treatment (i.e., prior enzalutamide in abiraterone-treated patients and vice versa). The study was approved by the local Ethics committee (2007-467-f-S and 2016–585-f-S) and all patients provided written informed consent. The study was conducted with provisions of the Declaration of Helsinki.

Study Design

This study aimed to evaluate the correlation of baseline CTC status (- vs. +) and AR-V7 status (- vs. +) to predict clinical benefit and outcome under therapy with ARTAs. Patients were asked to provide peripheral blood samples for CTC analysis at baseline. Enzalutamide was administered at 160 mg once daily, and abiraterone was administered at 1,000 mg once daily (with prednisone or prednisolone 5 mg bidaily). Follow-up patient visits were scheduled every 1 to 2 months.

Therapy with enzalutamide or abiraterone was continued until PSA progression, clinical or radio-graphic progression, whichever occurred first, or unmanageable drug-related toxicity.

Biomarker positive subgroup stratification based on mRNA copy numbers

To dissect absolute mRNA copy numbers for both KLK3-PSA and AR-V7, dsDNA oligonucleotides containing mRNA region detected by TaqMan Assays were diluted at known copy numbers and run in a qPCR to obtain a standard curve. Ct values obtained by qPCR using TaqMan assays for both KLK3-PSA and AR-V7 were converted to absolute mRNA copy numbers using this standard curve (Fig. S2). Status is presented as absolute copy numbers per 5ml blood sample.

For comparison of KLK3-PSA and AR-V7 mRNA copy numbers, biomarker positive patients were divided into responder (R) and non-responder (N) according to PSA 50 response, PFS and OS. Either the median PFS and OS or the upper limit of the CI of PFS and OS were used as the cut-off for a response in terms of PFS or OS. All patients with a time above the cut-off were assigned to the responder group irrespectively of occurrence of the event. Patients with an event and a time below the cut-off were considered non-responders. We excluded patients without an event and a time below the cut-off, as the follow-up time was not sufficient for a reliable classification.

Statistical Analyses

The statistical assessment was performed with the help of SPSS-Statistics V28.0 (IBM Inc., Armonk, NY) and Prism 8 V8.4.3 (GraphPad Software, LLC., San Diego, CA). The descriptive statistics are reported as medians with interquartile ranges (IQR) for continuous variables and as frequencies and populations for categorical variables.

PSA response rates were analyzed for CTC- and CTC+ patients as well as AR-V7- and AR-V7+ patients and Fisher’s exact test was used for statistical comparison. Outcomes were analyzed for CTC-, CTC+, AR-V7- and AR-V7+ patient cohorts. The CTC+ cohort was further subdivided into CTC+/AR-V7- and CTC+/AR-V7+ groups. Additionally, AR-V7- patients were stratified into AR-V7-/CTC- and AR-V7-/CTC+ cohorts. Time-to-event outcomes (PFS and OS) were evaluated performing Kaplan-Meier analysis, and survival-time differences were compared via log-rank test.

Comparisons of responder and non-responder regarding mRNA copy number were evaluated with Mann-Whitney test and comparisons of advanced stage criteria were statistically analyzed using t test. Chi-square was applied for testing distribution of CTC+ and AR-V7+ patients in different therapy lines.
