# Supplementary material for: Comparison of circulating tumor cells and AR-V7 as clinical biomarker in metastatic castration-resistant prostate cancer patients
Source: Sci Rep. 2022 Jul 13;12:11846. doi: 10.1038/s41598-022-16094-6 (PMC9279395; doi:10.1038/s41598-022-16094-6)
Supplement: Supplementary file 5 — Supplementary Tables. [file 41598_2022_16094_MOESM5_ESM.docx]

Table S1: Univariate analyses of biomarkers for PFS and OS in 65 mCRPC-patients on abiraterone or enzalutamide therapy (overall cohort)

Abbreviations: abi: abiraterone; ALP: alkaline phosphatase; AR-V7: androgen receptor splice variant 7; CI: confidence interval; CTC: circulating tumor cell; ECOG: eastern co-operative oncology group; enza: enzalutamide; Hb: hemoglobin; HR: hazard ratio; LDH: lactate dehydrogenase; PSA: prostate specific antigen

|  | **Progression Free Survival** | | **Overall Survival** | |
| --- | --- | --- | --- | --- |
| **Variable** | **HR (95% CI)** | **p** | **HR (95% CI)** | **p** |
| **Gleason-Score ≥ 8**  **No**  **Yes** | 1 (reference)  0.946 (0.476-1.877) | 0.873 | 1 (reference)  0.999 (0.536-1.863) | 0.999 |
| **Prior docetaxel**  **No**  **Yes** | 1 (reference)  1.077 (0.570-2.035) | 0.820 | 1 (reference)  1.693 (0.926-3.097) | 0.087 |
| **Prior abi or enza**  **No**  **Yes** | 1 (reference)  3.742 (1.892-7.401) | **<0.001** | 1 (reference)  3.772 (2.051-6.937) | **<0.001** |
| **Bone metastases**  **No**  **Yes** | 1 (reference)  1.937 (0.885-4.239) | 0.098 | 1 (reference)  2.337 (1.082-5.046) | **0.031** |
| **Visceral metastases**  **No**  **Yes** | 1 (reference)  1.441 (0.567-3.664) | 0.443 | 1 (reference)  2.295 (0.803-6.554) | 0.121 |
| **LDH elevated at baseline**  **No**  **Yes** | 1 (reference)  1.388 (0.766-2.517) | 0.280 | 1 (reference)  1.472 (0.836-2.590) | 0.180 |
| **ALP elevated at baseline**  **No**  **Yes** | 1 (reference)  1.806 (0.986-3.310) | 0.056 | 1 (reference)  2.294 (1.259-4.180) | **0.007** |
| **Hb** $\boldsymbol{\leq}$ **12 at baseline**  **No**  **Yes** | 1 (reference)  2.442 (1.198-4.977) | **0.014** | 1 (reference)  6.893 (3.406-13.949) | **<0.001** |
| **PSA doubling time < 3 months**  **No**  **Yes** | 1 (reference)  1.808 (0.993-3.292) | 0.053 | 1 (reference)  1.475 (0.833-2.611) | 0.183 |
| **Absence of PSA decline** $\boldsymbol{\geq}$ **50%**  **No**  **Yes** | 1 (reference)  3.930 (2.028-7.614) | **<0.001** | 1 (reference)  2.254 (1.265-4.017) | **0.006** |
| **CTC positivity**  **No**  **Yes** | 1 (reference)  3.966 (1.517-10.370) | **0.005** | 1 (reference)  5.243 (1.856-14.812) | **0.002** |
| **AR-V7 positivity**  **No**  **Yes** | 1 (reference)  2.685 (1.436-5.020) | **0.002** | 1 (reference)  2.949 (1.627-5.345) | **<0.001** |

Table S2: Univariate analyses of biomarkers for PFS and OS in 54 mCRPC-patients on abiraterone or enzalutamide therapy (CTC+ cohort)

Abbreviations: abi: abiraterone; ALP: alkaline phosphatase; AR-V7: androgen receptor splice variant 7; CI: confidence interval; ECOG: eastern co-operative oncology group; enza: enzalutamide; Hb: hemoglobin; HR: hazard ratio; LDH: lactate dehydrogenase; PSA: prostate specific antigen

|  | **Progression Free Survival** | | **Overall Survival** | |
| --- | --- | --- | --- | --- |
| **Variable** | **HR (95% CI)** | **p** | **HR (95% CI)** | **p** |
| **Gleason-Score ≥ 8**  **No**  **Yes** | 1 (reference)  0.847 (0.396-1.811) | 0.669 | 1 (reference)  0.944 (0.486-1.832) | 0.864 |
| **Prior docetaxel**  **No**  **Yes** | 1 (reference)  1.380 (0.702-2.714) | 0.350 | 1 (reference)  3.128 (1.530-6.396) | **0.002** |
| **Prior abi or enza**  **No**  **Yes** | 1 (reference)  2.653 (1.333-5.278) | **0.005** | 1 (reference)  2.522 (1.349-4.715) | **0.004** |
| **Bone metastases**  **No**  **Yes** | 1 (reference)  0.576 (0.222-1.493) | 0.256 | 1 (reference)  0.958 (0.403-2.276) | 0.922 |
| **Visceral metastases**  **No**  **Yes** | 1 (reference)  1.728 (0.603-4.947) | 0.308 | 1 (reference)  2.898 (0.838-10.015) | 0.093 |
| **LDH elevated at baseline**  **No**  **Yes** | 1 (reference)  1.322 (0.701-2.494) | 0.388 | 1 (reference)  1.660 (0.908-3.037) | 0.100 |
| **ALP elevated at baseline**  **No**  **Yes** | 1 (reference)  1.259 (0.667-2.376) | 0.477 | 1 (reference)  1.665 (0.897-3.090) | 0.106 |
| **Hb** $\boldsymbol{\leq}$ **12 at baseline**  **No**  **Yes** | 1 (reference)  1.694 (0.823-3.488) | 0.152 | 1 (reference)  5.144 (2.500-10.581) | **<0.001** |
| **PSA doubling time < 3 months**  **No**  **Yes** | 1 (reference)  2.001 (1.044-3.837) | **0.037** | 1 (reference)  2.037 (1.065-3.896) | **0.032** |
| **Absence of a PSA decline** $\boldsymbol{\geq}$ **50%**  **No**  **Yes** | 1 (reference)  2.815 (1.429-5.544) | **0.003** | 1 (reference)  1.581 (0.869-2.877) | 0.134 |
| **AR-V7 positivity**  **No**  **Yes** | 1 (reference)  1.797 (0.918-3.519) | 0.087 | 1 (reference)  1.969 (1.046-3.707) | **0.036** |

Table S3: Univariate analyses of biomarkers for PFS and OS in 32 mCRPC-patients on abiraterone or enzalutamide therapy (AR-V7- cohort)

Abbreviations: abi: abiraterone; ALP: alkaline phosphatase; CI: confidence interval; CTC: circulating tumor cell; ECOG: eastern co-operative oncology group; enza: enzalutamide; Hb: hemoglobin; HR: hazard ratio; LDH: lactate dehydrogenase; PSA: prostate specific antigen

|  | **Progression Free Survival** | | **Overall Survival** | |
| --- | --- | --- | --- | --- |
| **Variable** | **HR (95% CI)** | **p** | **HR (95% CI)** | **p** |
| **Gleason-Score ≥ 8**  **No**  **Yes** | 1 (reference)  0.796 (0.298-2.131) | 0.650 | 1 (reference)  0.914 (0.365-2.286) | 0.847 |
| **Prior docetaxel**  **No**  **Yes** | 1 (reference)  0.602 (0.173-2.091) | 0.425 | 1 (reference)  1.082 (0.355-3.296) | 0.889 |
| **Prior abi or enza**  **No**  **Yes** | 1 (reference)  5.377 (1.563-18.501) | **0.008** | 1 (reference)  2.570 (0.838-7.882) | 0.099 |
| **Bone metastases**  **No**  **Yes** | 1 (reference)  2.040 (0.712-5.840) | 0.184 | 1 (reference)  2.134 (0.697-6.533) | 0.184 |
| **Visceral metastases**  **No**  **Yes** | 1 (reference)  1.568 (0.452-5.435) | 0.478 | 1 (reference)  2.563 (0.539-12.192) | 0.237 |
| **LDH elevated at baseline**  **No**  **Yes** | 1 (reference)  1.291 (0.508-3.282) | 0.591 | 1 (reference)  1.216 (0.487-3.036) | 0.675 |
| **ALP elevated at baseline**  **No**  **Yes** | 1 (reference)  1.345 (0.478-3.784) | 0.574 | 1 (reference)  2.357 (0.887-6.265) | 0.086 |
| **Hb ≤ 12 at baseline**  **No**  **Yes** | 1 (reference)  3.277 (0.854-12.578) | 0.084 | 1 (reference)  16.331 (3.264-81.703) | **<0.001** |
| **PSA doubling time < 3 months**  **No**  **Yes** | 1 (reference)  1.927 (0.751-4.941) | 0.172 | 1 (reference)  1.165 (0.457-2.969) | 0.749 |
| **Absence of a PSA decline ≥ 50%**  **No**  **Yes** | 1 (reference)  4.434 (1.547-12.704) | **0.006** | 1 (reference)  1.720 (0.671-4.408) | 0.259 |
| **CTC positivity**  **No**  **Yes** | 1 (reference)  2.969 (1.020-8.643) | **0.046** | 1 (reference)  3.600 (1.162-11.151) | **0.026** |

Table S4: Multivariate analyses of biomarkers for PFS and OS in 65 mCRPC-patients on abiraterone or enzalutamide therapy (overall cohort)

Abbreviations: abi: abiraterone; AR-V7: androgen receptor splice variant 7; CI: confidence interval; CTC: circulating tumor cell; enza: enzalutamide; Hb: hemoglobin; HR: hazard ratio; PSA: prostate specific antigen

|  | **Progression Free Survival** | | **Overall Survival** | |
| --- | --- | --- | --- | --- |
| **Variable** | **HR (95% CI)** | **p** | **HR (95% CI)** | **p** |
| **CTC positivity**  **No**  **Yes** | 1 (reference)  2.653 (0.918-7.665) | 0.071 | 1 (reference)  3.320 (1.088-10.129) | **0.035** |
| **AR-V7 positivity**  **No**  **Yes** | 1 (reference)  1.478 (0.735-2.971) | 0.273 | 1 (reference)  1.488 (0.764-2.899) | 0.242 |
| **Prior abi or enza**  **No**  **Yes** | 1 (reference)  2.547 (1.237-5.242) | **0.011** | 1 (reference)  2.163 (1.133-4.133) | **0.019** |
| **Hb ≤ 12 at baseline**  **No**  **Yes** | 1 (reference)  1.440 (0.677-3.063) | 0.344 | 1 (reference)  4.377 (2.104-9.105) | **<0.001** |

Table S5: Multivariate analyses of biomarkers for PFS and OS in 54 mCRPC-patients on abiraterone or enzalutamide therapy (CTC+ cohort)

Abbreviations: abi: abiraterone; AR-V7: androgen receptor splice variant 7; CI: confidence interval; enza: enzalutamide; Hb: hemoglobin; HR: hazard ratio; PSA: prostate specific antigen

|  | **Progression Free Survival** | | **Overall Survival** | |
| --- | --- | --- | --- | --- |
| **Variable** | **HR (95% CI)** | **p** | **HR (95% CI)** | **p** |
| **AR-V7 positivity**  **No**  **Yes** | 1 (reference)  1.350 (0.665-2.741) | 0.406 | 1 (reference)  1.564 (0.803-3.046) | 0.189 |
| **Prior abi or enza**  **No**  **Yes** | 1 (reference)  1.643 (0.739-3.654) | 0.223 | 1 (reference)  1.915 (0.985-3.720) | 0.055 |
| **Absence of a PSA decline** $\boldsymbol{\geq}$ **50%**  **No**  **Yes** | 1 (reference)  2.036 (0.904-4.586) | 0.086 |  |  |
| **Hb ≤ 12 at baseline**  **No**  **Yes** |  |  | 1 (reference)  3.950 (1.861-8.383) | **<0.001** |

Table S6: Multivariate analyses of biomarkers for PFS and OS in 32 mCRPC-patients on abiraterone or enzalutamide therapy (AR-V7- cohort)

Abbreviations: abi: abiraterone; CI: confidence interval; CTC: circulating tumor cell; enza: enzalutamide; Hb: hemoglobin; HR: hazard ratio

|  | **Progression Free Survival** | | **Overall Survival** | |
| --- | --- | --- | --- | --- |
| **Variable** | **HR (95% CI)** | **p** | **HR (95% CI)** | **p** |
| **CTC positivity**  **No**  **Yes** | 1 (reference)  2.562 (0.851-7.714) | 0.094 | 1 (reference)  3.353 (1.073-10.474) | **0.037** |
| **Prior abi or enza**  **No**  **Yes** | 1 (reference)  4.166 (1.205-14.402) | **0.024** |  |  |
| **Hb ≤ 12 at baseline**  **No**  **Yes** |  |  | 1 (reference)  14.979 (2.995-74.907) | **<0.001** |
